# Identification and Functional Exploration of *BraGASA* Genes Reveal Their Potential Roles in Drought Stress Tolerance and Sexual Reproduction in *Brassica rapa* L. ssp. *pekinensis*

**DOI:** 10.3390/ijms25179643

**Published:** 2024-09-06

**Authors:** Yanting Zhao, Xinjie Sun, Jingyuan Zhou, Lixuan Liu, Li Huang, Qizan Hu

**Affiliations:** 1 Zhejiang Academy of Agricultural Sciences, Hangzhou 310021, China; zhaoyt@zaas.ac.cn; 2 College of Agriculture and Biotechnology, Zhejiang University, Hangzhou 310058, China; 22416199@zju.edu.cn (X.S.); lihuang@zju.edu.cn (L.H.); 3 Ziyun & Bifeng Community, Qiushi College, Zhejiang University, Hangzhou 310058, China; 3230100642@zju.edu.cn; 4 School of International Studies, Zhejiang University, Hangzhou 310058, China; 3220100194@zju.edu.cn

**Keywords:** *Brassica rapa*, *GASA*, gene family, bioinformatics, expression profile

## Abstract

Gibberellic acid-stimulated Arabidopsis sequences (*GASAs*) are a subset of the gibberellin (GA)-regulated gene family and play crucial roles in various physiological processes. However, the *GASA* genes in *Brassica rapa* have not yet been documented. In this study, we identified and characterized 16 *GASA* genes in Chinese cabbage (*Brassica rapa* L. ssp. *pekinensis*). Analysis of the conserved motifs revealed significant conservation within the activation segment of *BraGASA* genes. This gene family contains numerous promoter elements associated with abiotic stress tolerance, including those for abscisic acid (ABA) and methyl jasmonate (MeJA). Expression profiling revealed the presence of these genes in various tissues, including roots, stems, leaves, flowers, siliques, and callus tissues. When plants were exposed to drought stress, the expression of *BraGASA3* decreased notably in drought-sensitive genotypes compared to their wild-type counterparts, highlighting the potentially crucial role of *BraGASA3* in drought stress. Additionally, *BraGASAs* exhibited various functions in sexual reproduction dynamics. The findings contribute to the understanding of the function of *BraGASAs* and provide valuable insights for further exploration of the *GASA* gene function of the *BraGASA* gene in Chinese cabbage.

## 1. Introduction

Gibberellic acid-stimulated Arabidopsis sequences (*GASAs*) are a subclass of the gibberellic acid-regulated gene family widely distributed across the plant kingdom and involved in various aspects of plant growth, development, and physiological responses [1]. The *GASA* gene family member *GAST-1* was initially identified in a tomato mutant [2]. *GASA* genes encode low molecular weight proteins (ranging from 80 to 270 amino acids) and contain three distinct structural domains: an N-terminal signal peptide consisting of 18 to 29 amino acids and a highly variable region (comprising 7 to 31 amino acids). Variations in amino acid content and sequence length among family members are indicative of this diversity. The C-terminal *GASA* domain contains 60 amino acids and 12 conserved cysteine residues. These cysteine residues are responsible for forming the structural framework of these proteins and are critically important for biochemical activity and responses within the plant organism [3]. Moreover, previous studies have demonstrated that peptides without the GASA structural domain cannot perform these functions [4]. A few GASA proteins that have been functionally characterized are known to participate in numerous activities related to plant growth and development. GASA proteins play a role in the sexual reproductive processes of plants, particularly in the development of fruits and seeds, as well as in the induction of floral organ production. It is reported that *AtGASA5* suppresses flowering and *AtGASA4* promotes flowering [1,3]. The regulation of apple tree flowering may be aided by the *MdGASA* gene [5]. In the final phases of seed development, *VvGASA2* and *VvGASA7* show differential expression levels in grapes, suggesting a role in seed maturation [6]. Grain length in common wheat is influenced by *TaGASR7*-A1 under different farming conditions [7].

Moreover, GASA proteins contribute to the ability of plants to withstand biotic and abiotic stress. *SNAKIN*-1 and *SNAKIN*-2 (*StSN1* and *StSN2*) exhibit antifungal and antibacterial properties in potatoes [8]. The FaGAST2 protein exerts a direct role in the scavenging of reactive oxygen species in response to fungal infection [9]. When rubber plants come into contact with the fungus *Colletotrichum gloeosporioides*, the expression of *GASA* genes (*HbGASA*) is elevated [6]. *GsGASA1* is involved in soybean root growth suppression induced by chronic cold through the accumulation of *DELLA* genes [10]. In *Arabidopsis*, the *AtGASA5* gene has a negative regulatory effect on heat tolerance by regulating the translation of salicylic acid (SA) signals and accumulation of heat shock proteins [11]. By modifying ROS accumulation, *GASA14* regulates the ability of *Arabidopsis* plants to withstand abiotic stress [12]. The response of common beans to salt stress may be mediated by the *Pvul-GASA-1* gene [13]. Other studies have shown that GASA proteins influence hormone-related processes, including seed germination, flower development, stem elongation, root development, signal transduction, fruit development, ripening, and the growth of meristematic tissue.

Chinese cabbage is one of the most economically important *Brassica* species and is mainly cultivated as a vegetable crop worldwide. It not only underwent a whole-genome triplication (WGT) event after its divergence from *Arabidopsis* but also experienced recent genome duplications [14]. Due to the high degree of sequence similarity and conserved genome structures, most genes, pathways, and physiological processes are shared between Chinese cabbage and *Arabidopsis*. This close relationship allows many findings from *Arabidopsis* to be readily applied to Chinese cabbage. The WGT event in *B. rapa* offers a crucial reference for understanding the evolution of polyploid genomes and the dynamics of gene families. The draft reference genome of Chinese cabbage was first completed in 2011 [14]. Since then, it has undergone three major updates in 2017 [15], 2018 [16], and 2022 [17]. These updates utilized higher-depth long reads generated from third-generation sequencing (TGS), high-throughput chromosome conformation capture (Hi-C), and Pacific Biosciences (PacBio), respectively. Additionally, in 2021 [18], a pangenome with structural variations of 18 *B. rapa* accessions was published, further enhancing our understanding of its genetic diversity. While there has been significant progress in sequencing and assembling the genome, studies of functional genomics remain limited. This gap highlights the need for more focused research to fully understand the biological roles of genes in Chinese cabbage. Through gene family identification, the potential functions of genes can be inferred based on the functions of known families, which helps in understanding the role of each gene within the genome. In this study we employed bioinformatic techniques to identify the members of the *GASA* gene family in Chinese cabbage and conducted a comprehensive phylogenetic analysis. In addition, the transcriptional levels of *GASA* genes under drought stress and post-pollination were measured. This work establishes a scientific basis for future investigations into the functional features of Chinese cabbage.

## 2. Results

### 2.1. Identification and Physicochemical Characterization of BraGASA Family Genes

**Figure 1:**
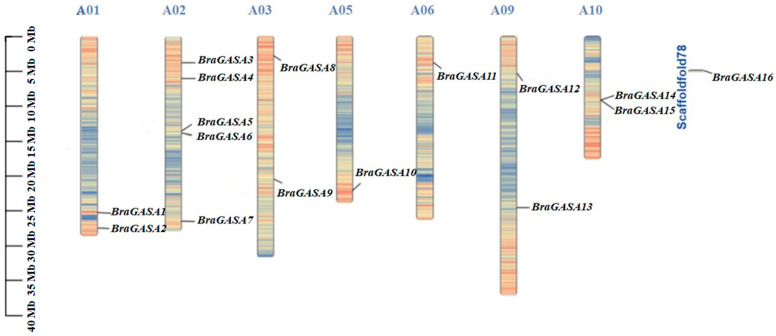
Chromosome distribution of *BraGASA* genes.

We identified 16 members of the *BraGASA* gene family and located them on chromosomes using bioinformatics. *BraGASA* genes were numbered sequentially based on their location on the chromosome (Table 1 and Figure 1). The number of members of the *BraGASA* gene family was similar to that in *Arabidopsis*. As shown in Figure 1, five *BraGASAs* were found on chromosome A02, followed by A01 (two genes), A03 (two genes), A09 (two genes), A10 (two genes), A05 (one gene), and A06 (one gene). *BraGASAs* were not found on chromosomes A04, A07, or A08, and *BraGASA16* was localized on the scaffold. The MW of the BraGASA proteins ranged from 7172.29 Da to 27,171.56 Da, and the theoretical isoelectric point ranged from 7.81 to 10.14. Among them, *BraGASA2* had the lowest isoelectric point (7.81), whereas *BraGASA8* had the highest (10.14) (Table 1).

### 2.2. Phylogenetic Relationships and Synteny

**Figure 2:**
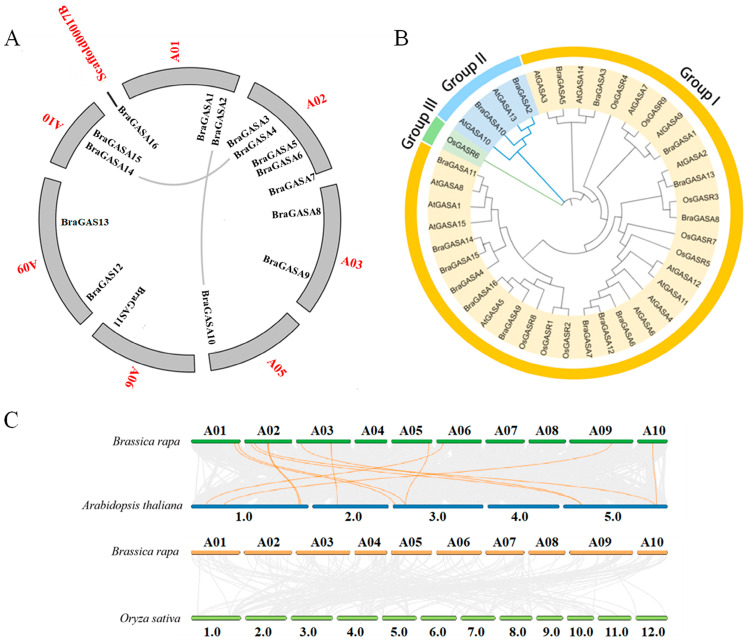
Chromosomal localization and phylogenetic analysis of *BraGASA*. (**A**) Gene distribution and repeated occurrences of *BraGASA*. The gray lines are the pairs of genes that are replicated in segments. (**B**) Phylogenetic trees for maximum likelihood analysis of *GASA* gene families in *B. rapa*, *Arabidopsis*, and rice. (**C**) Collinearity analysis of *GASA* gene in *B. rapa*, *Arabidopsis*, and rice. Interspecies pairs of GASA protein kinase homologous genes are linked with orange lines.

Segment duplication and tandem repeats are considered the main causes of gene family expansion. As shown in Figure 2A, two chromosomal segment duplications were detected in *BraGASAs* and no tandem repeats were detected, indicating that chromosomal segment duplications may be the main cause of *BraGASA* gene expansion in the Chinese cabbage genome.

MEGA (version 11.0) was used to perform multiple sequence alignment of *GASA* family genes in Chinese cabbage, the two model plant species, *Arabidopsis* and rice, then a phylogenetic tree was constructed. The results showed that *GASA* family genes in Chinese cabbage, *Arabidopsis*, and rice were divided into three clusters (Groups), of which *GASA* genes in Group I were the most abundant, including 14 *AtGASAs*, 15 *BraGASAs,* and *8 OsGASRs,* while group III had the lowest number, which included only one *OsGASR* (Figure 2B).

To further clarify the origin and evolution of *BraGASA*, the comparative collinear relationships between Chinese cabbage, *Arabidopsis,* and rice were analyzed. The *BraGASA* and *AtGASA* gene family had 12 collinear pairs, while no collinear relationship was observed between *BraGASA* and *OsGASR* (Figure 2C). These findings suggest a close evolutionary relationship and functional similarity between Chinese cabbage and *Arabidopsis*.

### 2.3. Gene Structure and Protein Domain

**Figure 3:**
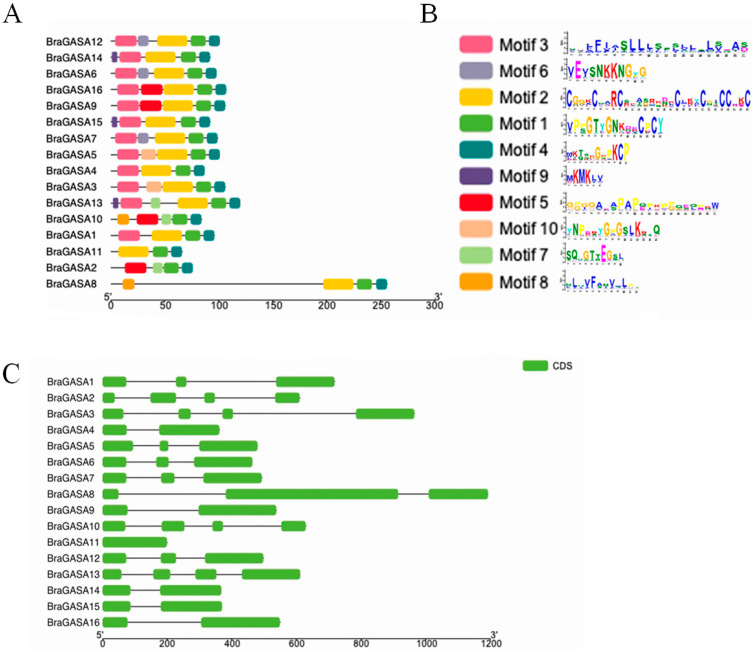
Gene structure and conserved motifs of *BraGASA*. (**A**) Composition and distribution of conserved motifs in *BraGASA*. (**B**) Motif logo of motif 1~motif 10. (**C**) Exon-intron structure of *BraGASA*. Green boxes indicate exons and lines connecting exons are introns.

Protein domain analysis was performed using TBtools and the NCBI CDD tool, which obtained ten conserved motifs in the *BraGASA* family (Figure 3A,B). The number and distribution of motifs varied among genes, ranging from 3 to 6, with all *BraGASA* family members containing motif 1 and motif 4, and all except *BraGASA2* including motif 2. Notably, the distance between motif 8 and motif 2 in *BraGASA8* was significantly larger compared to other genes. Additionally, the distribution of GASA protein motifs in Chinese cabbage was relatively even, indicating minimal variation in motif dispersion among family members.

TBtools was used to map the structure of the 16 *GASA* genes in *B. rapa*. The analysis revealed minimal variation in the number of exons among the *BraGASA* gene family, with counts ranging from one (in *BraGASA11*) to four (in *BraGASA2, BraGASA3, BraGASA10,* and *BraGASA13*). Most family members contained three exons (Figure 3C).

### 2.4. Analysis of Promoter Cis-Regulatory Elements of BraGASAs

**Figure 4:**
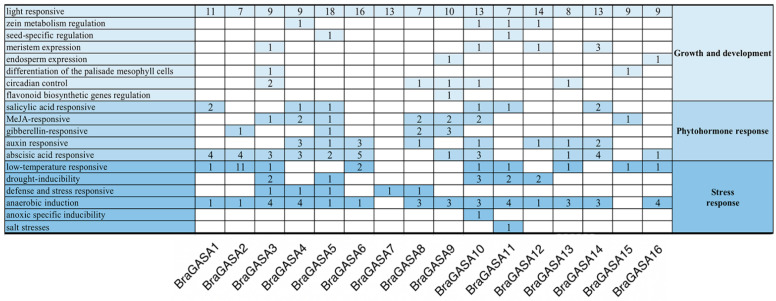
*BraGASA* promoter region cis-element analysis: the number of cis-elements in each gene is expressed numerically.

Gene transcription is primarily controlled by the recognition and binding of DNA sequence motifs in cis-regulatory regions by transcription factors, which activate or repress transcription to mediate responses to changes in the external environment [2]. To investigate the gene function and transcriptional regulatory mechanisms of *BraGASA*, we utilized the 2000 bp upstream region of the *BraGASA* gene coding sequence for cis-element prediction. These elements are involved in growth and development processes, phytohormones, and stress responses (Figure 4).

Promoter element analysis revealed that all *BraGASA* genes contain light-response elements, with their numbers significantly higher compared to other elements. Among the 16 family members, 9 contained low-temperature and drought-inducibility response elements, 14 contained anaerobic induction response elements, and 11 contained ABA response elements. Notably, *BraGASA2* had a higher number of low-temperature response elements, while *BraGASA3* featured more ABA-responsive, drought-inducible, and anaerobic induction elements. Additionally, the *BraGASA* family members also possessed elements related to meristem expression, defense and stress responses, biological clock regulation, and protein metabolism. This suggested that the *BraGASA* family played a broad role in the growth, development, and stress responses of Chinese cabbage.

### 2.5. Analysis of BraGASA Protein Tertiary Structure

**Figure 5:**
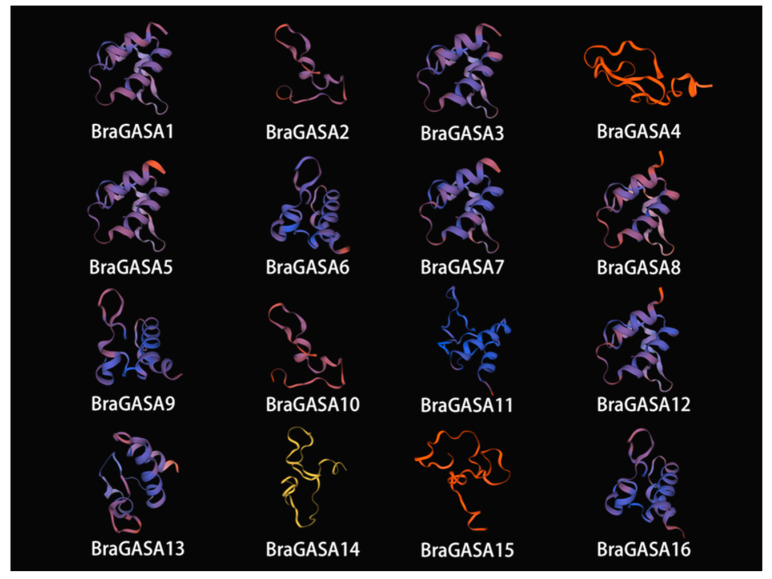
Predicted 3-D structures of GASA proteins.

The tertiary structure of proteins is formed by the further curling and folding of their secondary structure. Computer analysis was used to determine the subcellular location and structural properties of the proteins. The results revealed that the predicted protein structure of BraGASA proteins included α helices and β sheets, with α helices being the most abundant and β sheets comparatively fewer (Figure 5). Among them, *BraGASAS4*, *BraGASA14*, and *BraGASA15* exhibited significantly fewer α helices in their tertiary structure than the other proteins.

### 2.6. Gene Expression Analysis of the BraGASAs

**Figure 6:**
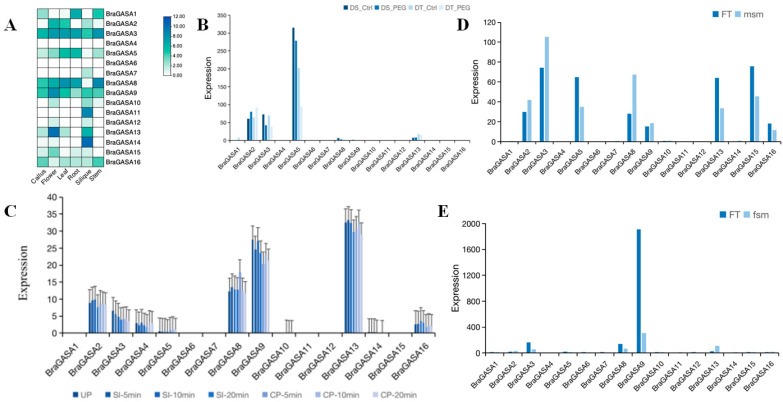
(**A**) Analysis of *GASA* gene transcript levels in different tissues of *B. rapa*. The darker color indicates higher expression. All values are logarithmically transformed. (**B**) Analysis of *GASA* gene transcriptome data in *B. rapa* under drought stress. Drought-tolerant (DT) and drought-sensitive (DS) plants were treated with PEG and expressed as DT-PEG and DS-PEG, respectively. (**C**) The qRT-PCR verification of expression levels of unpollinated (UP), self-pollinated (SI), and cross-pollinated (CP) plants. (**D**) Transcriptome analysis of male sterile mutant (msm) and wild-type (FT) *GASA* genes. (**E**) Transcriptome analysis of female sterile mutant (fsm) and wild-type (FT) *GASA* genes.

To explore the potential function of *BraGASAs*, we analyzed their expression levels in various plant tissues, including calluses, flowers, leaves, roots, siliques, and stems (Figure 6A). Fourteen genes (*BraGASA4* and *BraGASA6* were the exceptions) were expressed in at least one tissue, and six genes were expressed in all six tissues. Moreover, the gene expression of *BraGASAs* exhibited significant tissue specificity. *BraGASA11* and *BraGASA14* were expressed only in siliques, while the expression level of *BraGASA13* in flowers was almost 13 times higher than that in siliques. Most *BraGASA* genes showed significant expression differences across tissues, with high expression in siliques followed by flowers. Expression was generally lower in roots, stems, and leaves, and lowest in the calluses. These distinct tissue expression patterns suggested that *BraGASA* genes have specialized roles at different stages of Chinese cabbage growth and development.

### 2.7. Analysis of Transcriptional Expression under Drought Stress

Transcriptome analysis of drought-sensitive and drought-tolerant Chinese cabbage showed that the expression of *BraGASA2* was significantly increased in drought-sensitive plants (Figure 6B). In contrast, the expression of *BraGASA3* and *BraGASA5* decreased significantly after drought stress in drought-sensitive plants. This is in line with promoter element analysis, which identified two drought-induced elements in *BraGASA3* and one in *BraGASA5* (Figure 4), highlighting their response to drought in sensitive plants. Conversely, *BraGASA2* expression was elevated in the drought-tolerant plants under drought stress, while *BraGASA3* and *BraGASA5* expression significantly decreased. Notably, *BraGASA13* expression increased in drought-sensitive plants but decreased in drought-tolerant plants, indicating the various roles of *BraGASAs* upon drought stress.

### 2.8. Analysis of Sexual Reproduction-Related Expression Profiling of BraGASAs

Chinese cabbage is a typical self-incompatible crop, and the expression patterns during self-incompatible reactions were distinct from those during cross-pollination reactions. Here, RNA-seq was used to detect the gene expression levels in the stigmas of unpollinated (UP), self-pollinated (SI), and cross-pollinated (CP) plants at different times. Then qRT-PCR was used to verify the reliability of the RNA-seq results (Figure 6C). The relative expression levels of *BraGASA* genes in the stigma were significantly different under different pollination conditions. The expression of *BraGASA1, BraGASA6, BraGASA7, BraGASA11, BraGASA12,* and *BraGASA15* was not detected whereas the gene expression levels of *BraGASA5, BraGASA10,* and *BraGASA14* were low. Moreover, most *BraGASA* family members showed small differences in gene expression between SI and CP plants. In contrast, the expression levels of *BraGASA2* at 5 and 10 min of self-pollination were significantly higher than those of outcrossing. The expression levels of *BraGASA9* and *BraGASA16* at 5, 10, and 15 min of self-pollination were higher than those at outcrossing pollination, indicating that these genes may play an important role in the process of self-incompatibility in *B. rapa*.

Hybrid seed production using male sterile lines is an important method to induce heterosis in *B. rapa*. Transcriptome analysis of male sterile mutants (msm) and wild-type (FT) of GASA gene showed that the expression of *BraGASA2*, *BraGASA3*, *BraGASA8*, and *BraGASA9* in msm were significantly increased compared with that in FT, indicating that these genes are involved in stamen growth and development in *B. rapa* (Figure 6D). By contrast, the expression of *BraGASA5*, *BraGASA13*, *BraGASA15*, and *BraGASA16* in msm was significantly lower in msm than that in FT, which may imply that these genes may negatively regulate the growth and development of stamens.

Pistil development is a complex process, and female sterile mutants are ideal materials for screening and cloning genes involved in pistil development, as well as for understanding the regulatory mechanisms of flower development [19]. Analysis of the transcriptome data of female sterile mutants (fsm) and wild-type (FT) of *GASA* in *B. rapa* revealed that *BraGASA2* and *BraGASA13* expression was significantly increased in fsm. The level of *BraGASA13* significantly increased to 12.5-fold relative to FT, whereas *BraGASA9* expression was significantly decreased compared to FT (Figure 6E), suggesting that these genes may be associated with development of the pistil.

### 2.9. Functional Annotation and Protein Interaction Analysis

**Figure 7:**
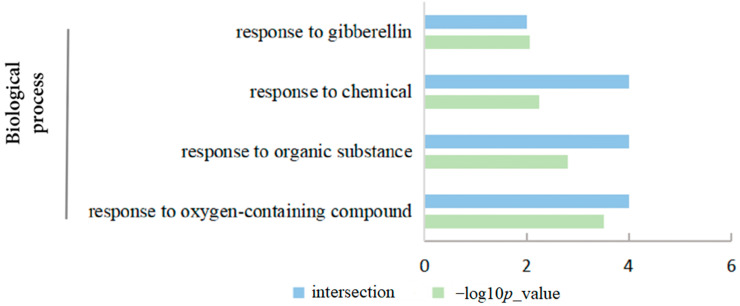
Gene ontology of *BraGASA* genes based on biological processes.

To further explore the function of *BraGASAs*, we analyzed the function of *BraGASAs* by GO annotations and enriched terms (including Biological Process (BP), Cellular Component (CC), and Molecular Function (MF)). The results showed that the *BraGASA* family was not enriched in GO-MF or GO-CC term. Four of the BraGASA proteins were involved in the response to oxygen-containing compounds (GO:1901700), organic substances (GO:0010033), and chemicals (GO:0042221), while two of these *BraGASA* gene were involved in the response to GA (GO:0009739) (Figure 7).

**Figure 8:**
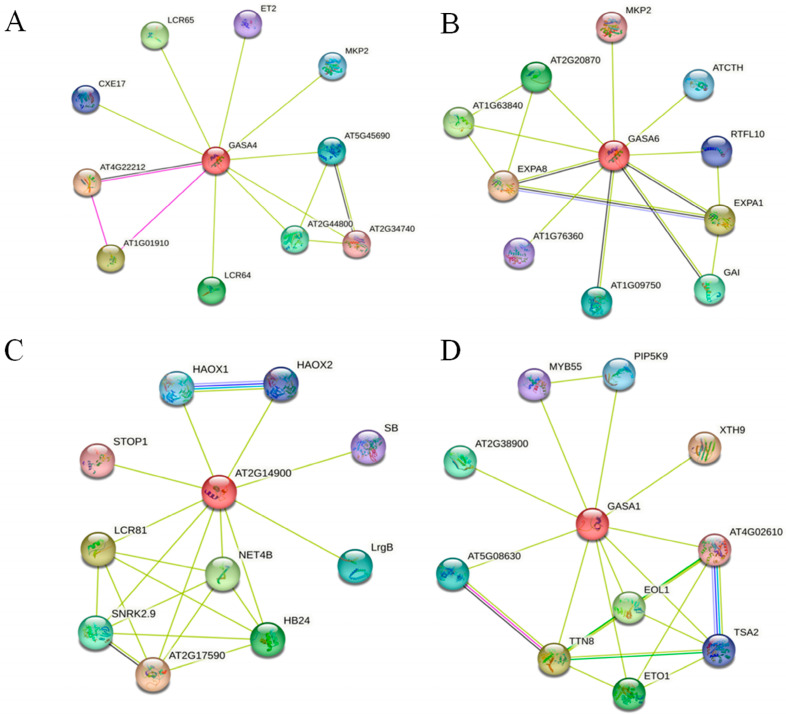
PPI network of Arabidopsis GASA protein kinases. Each node represents a protein and interacting proteins are connected by lines. Node size and filling color were positively correlated with degree centrality. Identified interacting proteins of GASA4 (**A**), GASA6 (**B**), AT2G14900 GASA7 (**C**), and GASA1 (**D**).

To fully understand biological phenomena, it is essential to consider the interaction networks between proteins. As the *BraGASA* genes are closely related to *AtGASA*, we performed PPI network analysis of AtGASA to further elucidate the function of BraGASA proteins. The PPI network identified associations between GASA proteins and several key proteins: CPL2 (which regulates plant growth, stress, and phytohormone responses), ERD15 (expressed under drought stress, light stress, and rhizobium treatment), LrgB (which plays a crucial role in chloroplast development and photorespiration), and SPL8 (which can increase plant biomass yield and sugar release). These associations suggested that GASA proteins may regulate various biological processes by interacting with these proteins. Additionally, both AtGASA4 (a homolog of BraGASA3) and AtGASA6 (a homolog of BraGASA2, BraGASA5) were linked to stress-related protein AtMKP2. Furthermore, AtGASA6 interacts with flowering-related ATCTH (also known as AtTZF1) (Figure 8). These findings underscored the significant and diverse roles of *BraGASA* genes in plant growth and stress responses.

## 3. Discussion

GA are critical in various aspects of plant growth and development, including seed germination, plant height, organ size, and reproductive processes. Recent research has increasingly focused on identifying and characterizing GA-responsive genes. Notably, the GA-stimulated transcript (*GAST1*) was characterized in the tomato fruit by Olszewski’s group [2]. *GASA* is a GA-regulated gene family from *Arabidopsis* related to the tomato *GAST1* gene [20]. In rice, *OsGSR1* (a member of the *GAST* gene family) shares significant similarity with its *Arabidopsis* counterpart, *AtGASA4*, with a 48% identity in their amino acid sequences. Additionally, *OsGASRs*, a group of rice GAST homologue genes, are related to *AtGASAs* and feature the GASA domain at their C-terminus [21]. GASA proteins significantly influence the production of plant floral organs, including seed development, fruit formation, responses to biotic and abiotic stressors, and hormone signal transduction. In this study we identified a total of 16 *BraGASA* genes in Chinese cabbage (Figure 1), which is more than the 15 found in *Arabidopsis* [5] and the 9 found in rice [5,22]. The MW of the BraGASA proteins ranged from 7172.29 Da to 27,171.56 Da, and the theoretical isoelectric point ranged from 7.81 to 10.14. BraGASA2 had the lowest isoelectric point (7.81), whereas BraGASA8 had the highest (10.14) (Table 1). All the identified *GASA* genes contained a conserved GASA domain and exhibited similar physicochemical characteristics. *GASA* genes are distributed across all chromosomes in Chinese cabbage (Figure 1), similar to their distribution in *Arabidopsis* [23]. 

GASA proteins significantly influence the production of plant floral organs, including seed development, fruit formation, responses to biotic and abiotic stressors, and hormone signal transduction [22]. In this study we identified a total of 16 *BraGASA* genes in Chinese cabbage (Figure 1), which is more than the 15 found in *Arabidopsis* [5] and the 9 found in rice [5,23]. The molecular weight (MW) of the BraGASA proteins ranged from 7172.29 Da to 27,171.56 Da, and the theoretical isoelectric point ranged from 7.81 to 10.14. BraGASA2 had the lowest isoelectric point (7.81), whereas BraGASA8 had the highest (10.14) (Table 1). All the identified *GASA* genes contained a conserved GASA domain and exhibited similar physicochemical characteristics. *GASA* genes are distributed across all chromosomes in Chinese cabbage (Figure 1), similar to their distribution in *Arabidopsis* [1]. *AtGASA*, *OsGASR*, and *BraGASA* were divided into three different groups based on phylogenetic analysis. There were 12 pairs of collinear interactions between the *BraGASA* and *AtGASA* of the model plant *Arabidopsis* (Figure 2A). However, no collinearity has been found between *OsGASR* and *BraGASA* (Figure 2B). These results indicate a reasonably close evolutionary link and functional similarity between *Arabidopsis* and Chinese cabbage. Previously, the *GASA* gene family has been widely investigated in different plant species and structural analysis of GASAs in various species revealed that the typical features of plant GASA proteins are conserved. Genes that are highly conserved across different species often have crucial and well-conserved functions that are essential for basic cellular processes. In the case of *BraGASA* genes, their conservation could indicate that they play fundamental roles in growth and development, such as regulating cell elongation, division, or response to environmental signals.

To further investigate the potential functions of the *BraGASA* gene family, we analyzed the cis-regulatory elements in Chinese cabbage (Figure 4). Among the 16 family members, 9 contained elements responsive to low-temperature and drought, 14 contained elements associated with anaerobic induction, and 11 contained ABA response elements, indicating that the *BraGASA* gene family likely plays a significant role in the growth, development, and photosynthesis of *B. rapa*. Notably, *BraGASA3* exhibited a higher number of ABA-responsive, drought-inducible, and anaerobic induction elements. This observation aligns with transcriptome data showing a significant decrease in *BraGASA3* expression in both drought-susceptible and drought-resistant plants following exposure to drought stress (Figure 8). In contrast, *BraGASA2* expression was elevated in both drought-tolerant and drought-sensitive plants, suggesting it might be crucial in the Chinese cabbage response to drought stress. However, the expression pattern of *BraGASA3* is opposite to that of *BraGASA2*, suggesting that different *GASA* family members may have distinct or even opposing functions. Of course, the data require more experimental validation, and the specific mechanisms need further investigation. Interestingly, this divergence in function among *GASA* genes has been noted in other studies. For example, overexpression of *AtGASA5* inhibited stem elongation and delayed flowering, whereas overexpression of *AtGASA6* promoted early flowering [1].The involvement of *GASA* genes in drought stress responses has been well documented. For instance, in common wheat (*Triticum aestivum*) [6], *GASA1* is strongly induced by drought, and two tomato *GASA* genes also show high expression levels under drought conditions [2]. Additionally, ectopic expression of *SmGASA4* was found to enhance drought resistance in plants [24]. In *Populus*, *GASA* genes are widely involved in drought stress responses, with homologs of *AtGASA1* being significantly up-regulated in drought-stressed *P. euphratica* leaves [25]. However, in *Arabidopsis*, *GASA1* is down-regulated under drought conditions, illustrating that even homologous genes from different species can exhibit varying expression patterns in response to drought stress [26]. Given these contrasting roles, it is essential to further investigate the *GASA* gene family to understand their functions in plant growth, development, and stress responses.

Previous studies have suggested that the *GASA* gene family exhibits tissue-specific expression patterns. For instance, in *Arabidopsis*, *GASA1* and *GASA2* are more highly expressed in flower buds and siliques [20], while *GASA4* is primarily expressed in meristematic regions [23]. Similarly, in rice, the *GASR9* gene is highly expressed in panicles [27]. Moreover, *GASA* genes tend to be more expressed in young tissues than in older ones [28]. In grapevine (*Vitis vinifera* L.), *GASA1* and *GASA2* are predominantly expressed in leaves, whereas *GASA9* and *GASA10* show high expression in fruits and seeds [29].Consistent with these findings, our study also revealed distinct expression patterns of *BraGASAs* in stems, flowers, and leaves (Figure 6A). Overall, tissue-specific expression patterns provide valuable insights into the functional roles of genes in different biological contexts. Genes expressed in specific tissues often contribute to the specialized functions of those tissues; for example, genes expressed in leaves might be involved in photosynthesis, while those in flowers might regulate reproductive processes. This understanding helps clarify how genes contribute to an organism’s overall physiology and adaptation to its environment. *BraGASA9* and *BraGASA13* exhibited relatively high expression in flowers compared to other tissues, suggesting their involvement in floral and seed development. This was further supported by transcriptome and qRT-PCR analysis, indicating that *BraGASA9* and *BraGASA13* play crucial roles in the development of stamens and pistils (Figure 6). To be honest, the findings are drawn from bioinformatics and omics data, and thus still need further experimental validation. Male sterility and self-incompatibility are the main methods currently used for seed production in Chinese cabbage. These methods simplify the hybridization process and are suitable for large-scale commercial seed production. Understanding the development of stamens and pistils offers valuable insights into the physiological and molecular mechanisms of male sterility. This knowledge facilitates the selection and cultivation of sterile lines, enhances their stability and adaptability, and ultimately improves seed production efficiency.

## 4. Materials and Methods

### 4.1. Analysis of GASA Family Proteins: Gene Sequences and Physical and Chemical Properties

Gene names and accession numbers of *Arabidopsis GASA* family proteins were acquired through the literature [30]. Subsequently, the protein sequences of the *Arabidopsis GASA* gene family were obtained from TAIR (https://www.arabidopsis.org, accessed on 4 November 2023). Genome-wide protein sequences and gff3 files of *B. rapa* were downloaded from PlantGDB (http://www.plantgdb.org/, accessed on 3 January 2024) (GenBank: GCA_008629595.1) to establish a local BLAST database. The protein sequences of the *Arabidopsis GASA* gene family were aligned with the putative genes in *B. rapa* using a local BLAST analysis with parameters set at an E-value threshold of less than 1 × 10^−10^ and an identity greater than 40%. The TBtools software (v1.120) was used to generate a structural diagram of *GASA* genes in *B. rapa* (*BraGASA* genes). Protein sequences containing the canonical GASA structural domain were identified by screening the NCBI Conserved Domain Database (https://www.ncbi.nlm.nih.gov/structure/cdd/wrpsb.cgi/, accessed on 8 January 2023) and SMART (SMART: Main page (embl.de), accessed on 14 January 2024). These results were consolidated by removing the duplicate genes and genes lacking the typical GASA structural domain. Sequences with incomplete open reading frames were manually corrected, and these sequences were renumbered based on the position of the family members on the chromosomes. Subsequently, the physical and chemical properties of the *BraGASA* gene family, including protein molecular weight (MW) and theoretical isoelectric point (pI), were predicted using the online tool ExPaSy (http://web.expasy.org/, accessed on 14 January 2024) and the software program TBtools, with default parameters.

### 4.2. Chromosomal Location, Phylogenetic Tree Construction, and Analysis of Collinearity Relationship

Genomic annotation information from the gff3 file of *B. rapa* was used to extract the genomic position of each *BraGASA* gene on individual chromosomes. Using MEGA 11.0, the amino acid sequences of the *BraGASA* gene family were aligned with the amino acid sequences of the *Arabidopsis GASA* gene family and rice *GASA* gene families through multiple alignments. Phylogenetic trees of the *GASA* gene families in *Arabidopsis*, rice, and *Brassica* were constructed using the Maximum Likelihood Estimate method. The bootstrap parameter was set to 1000, with all other parameters maintained at their default values for accuracy in the evolutionary tree analysis. The collinearity relationships between *B. rapa* and *Arabidopsis*, as well as the relationship between *B. rapa* and rice, were analyzed using the multiple linear scan toolkit (MCScanX) (http://chibba.pgml.uga.edu/mcscan2/, accessed on 30 February 2022) plugin with in TBtools. The results of this analysis were visually represented using the Circos plugin.

### 4.3. Prediction of Gene Structure and Conserved Motifs

The structure of the *BraGASA* genes was generated using TBtools. Conserved protein motifs within *BraGASA* were predicted using MEME 26 (http://meme-suite.org/, accessed on 11 November 2023). Furthermore, the Multiple Sequence Alignment function within DNAMAN (v6.0) was employed to align *BraGASA* sequences, facilitating the analysis of conserved amino acid positions within the proteins.

### 4.4. Analysis of Cis-Acting Elements

Gene family sequences comprising the upstream 2000 bp from the initiation codon of *BraGASA* family members were retrieved and downloaded by querying Ensembl Plants27 (http://plants.ensembl.org/index.html, accessed on 6 February 2024). Subsequently, the PlantCARE [1] online tool (http://bioinformatics.psb.ugent.be/webtools/plantcare/html/, accessed on 8 February 2024) was used with default parameters to predict and analyze the cis-acting element features within these sequences.

### 4.5. Prediction of Three-Dimensional Structures of the Proteins

Using the SWISS-MODEL online tool (https://swissmodel.expasy.org/interactive, accessed on 17 February 2024) provided by ExPaSy, three-dimensional structural homology modeling was performed for the proteins of the *BraGASA* gene family.

### 4.6. Protein–Protein Interaction Analysis and Gene Ontology Enrichment Analysis

The STRING online platform (http://cn.string-db.org, accessed on 25 February 2024) [31], with default parameters, was used to predict protein–protein interaction (PPI). Subsequently, Cytoscape v3.9.1 was employed to construct the interaction network. The online resource DAVID (http://david.ncifcrf.gov, accessed on 9 March 2024) was used to conduct Gene Ontology (GO) enrichment analysis for *BraGASA* genes based on functional similarities using default parameters. The resulting GO annotation data were processed and visually presented using Microsoft Excel 2022.

### 4.7. Analysis of Gene Expression of GASA in B. Rapa

Transcriptome data for distinct tissues of *B. rapa*, including male sterile mutants, female sterile mutants, drought-tolerant tissues, and drought-sensitive tissues, were acquired from the Brassicaceae Database (http://brassicadb.cn/ accessed on 15 March 2024). The accession numbers for these datasets are GSE43245 [25], GSE125485 [26], GSE147438 [27], and GSE73963 [28]. Subsequently, data visualization was conducted using the HeatMap plugin in TBtools and Microsoft Excel 2022 to generate graphical representations.

Unpollinated stigmas of *B. rapa* were collected at 5, 10, and 20 min after both self-pollination (SI) and cross-pollination (CP), and promptly preserved in liquid nitrogen. Each sample was collected in triplicate. Transcriptome sequencing (Table S1) was conducted using an Illumina NovaSeq 6000 (San Diego, CA, USA) platform at BioMarker Technologies (Beijing, China). Following sequencing, read counts and transcript lengths within the samples were normalized. Transcript abundance was quantified as fragments per kilobase of transcripts per million mapped reads (FPKM), and *BraGASA*-related gene expression data plots were generated using Microsoft Excel 2022.

The total RNA of samples was extracted using Plant Total RNA Isolation Kit (Sangon Biotech, Shanghai, China). The first cDNA strand was synthesized by the PrimeScript™RT reagent Kit with gDNA Eraser (Takara, Dalian, China). The expression of the gene encoding *Braactin-7* was used as an internal expression control. Real-time PCR was performed with the CFX96 real-time PCR machine (BIO-RAD, Berkeley, CA, USA) using TransStart^®^ Top Green qPCR SuperMix (TransGen, Beijing, China). The 2^−ΔΔCT^ Ct method was used to calculate the relative gene expression level across the samples.

## 5. Conclusions

**Figure 9:**
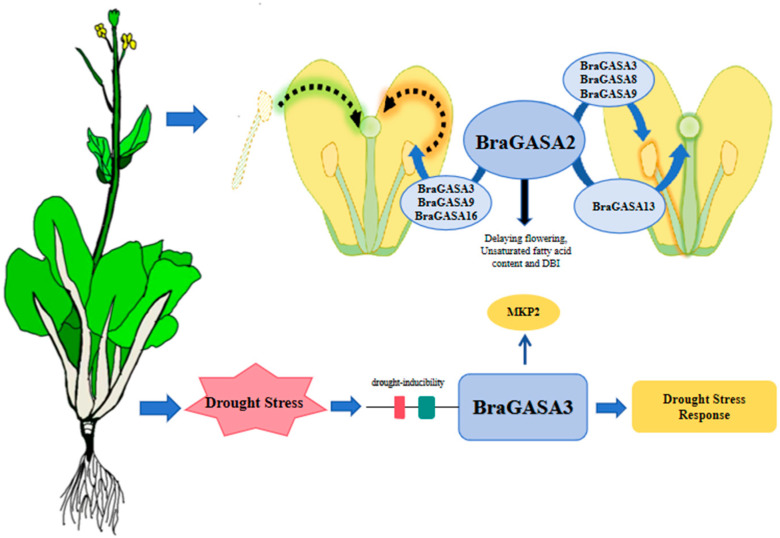
*GASAs* involved in response to drought stress and sexual reproduction in Chinese cabbage.

In summary, we identified 16 *BraGASA* genes in Chinese cabbage plants. Our comprehensive analysis, which included assessments of chromosomal localization, cis-regulatory elements, tissue-specific expression profiles, abiotic stress tolerance, and involvement in sexual reproductive processes, provided compelling evidence underscoring the potential pivotal role of *BraGASA2* in the regulation of sexual reproduction and the role of *BraGASA3* in drought stress tolerance in Chinese cabbage (Figure 9), for which further experiments and studies are needed to validate. Our funding establishes a scientific basis for future investigations into the functional features of *BraGASAs*, which might facilitate the selection and cultivation of sterile lines, thus enhancing their stability and adaptability, and ultimately improving the seed production efficiency of Chinese cabbage.

## Figures and Tables

**Table 1:** Basic information of the *BraGASA* gene family.

Gene ID	Gene Name	Chromosome	pI	MW (Da)	Protein Length (aa)	*A.thaliana* ID	*A.thaliana* Name
Bra021407	*BraGASA1*	A01:25317858:25318576	9.46	10,760.72	291	AT3G02885	AtGASA5
Bra034095	*BraGASA2*	A01:27475061:27475671	7.81	8400.38	231	AT1G74670	AtGASA6
Bra023513	*BraGASA3*	A02:3715274:3716239	9.36	11,704.76	321	AT5G15230	AtGASA4
Bra020281	*Br4GASA4*	A02:6005564:6005926	8.97	9647.36	264	AT5G59845	AtGASA10
Bra008162	*BraGASA5*	A02:13527439:13527919	8.95	11,443.45	306	AT1G74670	AtGASA6
Bra008222	*BraGASA6*	A02:13905515:13905979	9	10,578.39	297	AT1G75750	AtGASA1
Bra029227	*BraGASA7*	A02:26446073:26446566	8.79	10,558.37	300	AT1G75750	AtGASA1
Bra006273	*BraGASA8*	A03:2778795:2779988	10.14	27,191.56	771	AT5G14920	AtGASA13
Bra013115	*BraGASA9*	A03:20378333:20378871	8.86	11,232.3	321	AT2G14900	AtGASA7
Bra029820	*BraGASA10*	A05:22152528:22153157	9.61	9217.66	255	AT3G10185	AtGASA15
Bra019917	*BraGASA11*	A06:3722606:3722806	8.48	7172.29	201	AT2G39540	AtGASA8
Bra038550	*BraGASA12*	A09:5247947:5248445	8.99	10,838.64	306	AT1G75750	AtGASA1
Bra024530	*BraGASA13*	A09:24582390:24583002	9.1	13,187.23	363	AT1G22690	AtGASA9
Bra002526	*BraGASA14*	A10:9052766:9053136	8.62	10,241.93	279	AT5G59845	AtGASA10
Bra002525	*BraGASA15*	A10:9057487:9057855	8.75	10,229.9	279	AT5G59845	AtGASA10
Bra039830	*BraGASA16*	Scaffold000178:118569:119118	8.6	11,378.4	324	AT2G14900	AtGASA7

## Data Availability

All the data that support the findings of this study are available in the paper and its Supplementary Materials published online.

## Supplementary Materials

The following supporting information can be downloaded at https://www.mdpi.com/article/10.3390/ijms25179643/s1.
