# Supplementary material for: Identification and Functional Exploration of BraGASA Genes Reveal Their Potential Roles in Drought Stress Tolerance and Sexual Reproduction in Brassica rapa L. ssp. pekinensis
Source: Int J Mol Sci. 2024 Sep 6;25(17):9643. doi: 10.3390/ijms25179643 (PMC11395553; doi:10.3390/ijms25179643)
Supplement: Supplementary file 1 [file ijms-25-09643-s001.zip › ijms-3173959-supplementary.pdf]

International Journal of *Molecular Sciences*

MDPI

Article

Identification and Functional Exploration of *BraGASA* Genes Reveal Their Potential Roles in Drought Stress Tolerance and Sexual Reproduction in *Brassica rapa* L. ssp. *pekinensis*

Yanting Zhao [1,†], Xinjie Sun [2,†], Jingyuan Zhou [3], Lixuan Liu [4], Li Huang [2] and Qizan Hu [1,*]

[1] Zhejiang Academy of Agricultural Sciences, Hangzhou 310021, China; zhaoyt@zaas.ac.cn
[2] College of Agriculture and Biotechnology, Zhejiang University, Hangzhou 310058, China; 22416199@zju.edu.cn (X.S.); lihuang@zju.edu.cn (L.H.)
[3] Ziyun & Bifeng Community, Qiushi College, Zhejiang University, Hangzhou 310058, China; 3230100642@zju.edu.cn
[4] School of International Studies, Zhejiang University, Hangzhou 310058, China; 3220100194@zju.edu.cn
* Correspondence: huqizan@sohu.com
† These authors contributed equally to this work.

Supplementary materials:

Table S1. Expression of BrGASA genes after SI and CP pollination (FPKM values).

Gene ID	UP	Sl-5min	S1-l0min	S1-20min	SC-5min	SC-10mm
Bra002525	0	0	0	0	0	0
Bra002526	0277274	0.105294333	0.061050333	0.045346333	0	0.196712333
Bra019917	0	0	0	0	0	0
Bra006273	12.27013633	13.575528	12.92040533	12.72393533	17.97833533	12.65468933
Bra023513	6.564548	5.612534333	4.838759667	3.891487333	4.033123	4.307546333
Bra038550	0	0	0	0	0	0
Bra013115	27.525489	24.59988967	27.07122667	23.59322367	20.264936	22.89924367
Bra024530	32.55948	33.35358167	32 37267633	29.73793833	30.657789	32.745668
Bra029227	0	0	0	0	0	0
Bra021407	0	0	0	0	0	0
Bra020281	2.921569	2.364536	3.047641667	2 294396333	1.806305667	3.049098333
Bra034095	8.826495667	9.592678667	9.858081667	7 647982333	8.905079	8.654913333
Bra008222	0	0	0	0	0	0
Bra029820	0	0	0	0	0	0
Bra039830	2.584109333	2.625966333	3.575586333	3.061362667	1.770568667	2 228994333
Bra008162	0 303994	0.456195	0.443544667	0.381970667	0.980761667	1.356636
